# Protein functional module identification method combining topological features and gene expression data

**DOI:** 10.1186/s12864-021-07620-3

**Published:** 2021-06-08

**Authors:** Zihao Zhao, Wenjun Xu, Aiwen Chen, Yueyue Han, Shengrong Xia, ChuLei Xiang, Chao Wang, Jun Jiao, Hui Wang, Xiaohui Yuan, Lichuan Gu

**Affiliations:** 1 School of Computer and Information, Anhui Agricultural University, Hefei, Anhui, 230036 China; 2 Department of Computer Science and Engineering, University of North Texas, Denton, TX, 76203 United States

**Keywords:** Protein complexes, Topological features, Gene expression data, Evolutionary clustering

## Abstract

**Background:**

The study of protein complexes and protein functional modules has become an important method to further understand the mechanism and organization of life activities. The clustering algorithms used to analyze the information contained in protein-protein interaction network are effective ways to explore the characteristics of protein functional modules.

**Results:**

This paper conducts an intensive study on the problems of low recognition efficiency and noise in the overlapping structure of protein functional modules, based on topological characteristics of PPI network. Developing a protein function module recognition method ECTG based on Topological Features and Gene expression data for Protein Complex Identification.

**Conclusions:**

The algorithm can effectively remove the noise data reflected by calculating the topological structure characteristic values in the PPI network through the similarity of gene expression patterns, and also properly use the information hidden in the gene expression data. The experimental results show that the ECTG algorithm can detect protein functional modules better.

## Background

More and more clustering algorithms are proposed to identify protein complexes with the constantly development of proteomics. Although many of those algorithms have been verified to have good performance [1–4], mining the complex only through the protein network itself will inevitably limit the effectiveness of its results, because the available protein data is incomplete due to the diversity of protein network structures and the complexity of data sources, and there is a certain amount of noise in protein networks. Therefore, other biological data such as fusion of gene expression provide new ideas for detecting protein functional modules [5, 6]. For example, Chin et al. [7] proposed method HUNTER to detect functional modules, this method firstly calculates the similarity value of high-throughput data (for example, calculating pairwise similarity of gene expression patterns from microarray data), then, detecting weak signals that cannot be distinguished with existing methods by using the network of genes or proteins and the similarity values between them and by applying network topological constraints to the expression data clusters, finding connected sub-networks (or modules) with highly similarity, which improves the effectiveness of compound identification. Although there are many ways to analyze the network and similar data separately [8–11], there is still a lot of room for development in the method of using two information sources for analysis.

We find that topological structure and attribute information are very effective in identifying protein complexes by analyzing the existing mainstream PPI network methods for identifying protein functional modules [12, 13], even though there are not much approaches take both information into consideration. Moreover, many algorithms for detecting protein functional modules use some special optimized attributes to find clusters, obviously, the process of detecting protein functional modules can be regarded as an optimization problem [14, 15]. Therefore, this paper proposes a new protein complex recognition algorithm. ECTG(Evolutionary Clustering Algorithm Based on Topological Features and Gene expression data for Protein Complex Identification). This method is based on evolutionary algorithm (EA), which effectively fuses protein topology and gene expression data. It has an advantage of dispensing with working under linear constraints like a typical numerical optimization problem. It can also find multiple solutions and be executed in parallel, so it can solve big data source problem quickly and efficiently. In order to verify the performance of ECTG, we conducted experiments on three real PPI network data sets [16–18]: DIP, Krogan, and Gavin. The used compound standard set was the CYC2008 data set. The experimental results show that the algorithm proposed in this paper has more obvious advantages in multiple indicators.

## Methods

### Similarity measure of gene expression patterns

Calculating the similarity between gene expression patterns (co-expression degree) by using gene expression data has an important guiding function in understanding the relationship between the corresponding proteins of the gene, and can help to identify whether different proteins have same or similar functions and whether they can be composed as protein complexes or functional modules. At present, there are multiple similarity measurement methods for different data types. Methods such as Euclidean distance, Cosine similarity and Pearson correlation coefficient are usually used to calculate the similarity of gene expression patterns.

#### (1) Euclidean distance

Euclidean distance is often used to measure the similarity of a pair of gene expression data, that is, a n-dimensional vector. If given the genes *u* and *v*, the Euclidean distance between *u* and *v* is shown in formula 1:

1 $$ {d_{euc}}(u,v) = {\left({\sum\limits_{j = 1}^{n} {{{({u_{j}} - {v_{j}})}^{2}}}} \right)^{1/2}}  $$

In above formula, *u*_*j*_ and *v*_*j*_ are the expression components of gene *u* and gene *v* in dimension *j*.

But Euclidean distance is not suitable for calculating similarity between gene expression patterns with different dimensions. Therefore, it must be standardized to meet the requirements as mean equal zero and variance equal one when using Euclidean distance to measure the similarity of gene expression data.

#### (2) Cosine similarity, formula 2 as follow:

2 $$ {}\cos (\theta) = \frac{{A \cdot B}}{{\left\| A \right\|\left\| B \right\|}} = \frac{{{\sum\nolimits}_{i = 1}^{n} {{A_{i}} \times {B_{i}}} }}{{\sqrt {{\sum\nolimits}_{i = 1}^{n} {{{({A_{i}})}^{2}}}} \times \sqrt {{\sum\nolimits}_{i = 1}^{n} {{{({B_{i}})}^{2}}}} }}  $$

The larger the cosine value, the greater the similarity of gene expression patterns. When the cosine similarity is one, the gene expression patterns are completely consistent.

#### (3) Pearson correlation coefficient:

PCC is also an extensive used method for calculating the similarity of gene expression data. Given a gene *u* and a gene *v*, the calculation formula of the Pearson correlation coefficient between the two genes is shown in formula 3:

3 $$ {r_{pea}}(u,v) = \frac{{\sum\limits_{j = 1}^{n} {({u_{j}} - \overline u)({v_{j}} - \overline v)} }}{{\sqrt {\sum\limits_{j = 1}^{n} {{{({u_{j}} - \overline u)}^{2}}}} \sqrt {\sum\limits_{j = 1}^{n} {{{({v_{j}} - \overline v)}^{2}}}} }}  $$

In above formula, the definition of ${\overline u}$ and ${\overline v}$ are as follow:

$$\overline u = \frac{1}{n}\sum\limits_{j = 1}^{n} {{u_{j}}},\qquad \overline v = \frac{1}{n}\sum\limits_{j = 1}^{n} {{v_{j}}} $$

Since the Pearson correlation coefficient is sensitive to outlier data, false positive data is likely occur in the results, giving higher similarity values to dissimilar gene pairs, which will cause errors in the results. To avoid that, this paper measures the similarity of gene pairs by calculating the Jackknife correlation coefficient. Given *n* gene expression data samples under different conditions, the expression value of gene *u* under condition *j* is expressed as *u*_*j*_, given gene *u* and gene *v*, the Jackknife correlation coefficient *GEC* between the two genes can be obtained by the following formula 4:

4 $$ GEC(u,v) = min\{ {r_{pea}}({u^{{\mathrm{(}}j{\mathrm{)}}}},{v^{{\mathrm{(}}j{\mathrm{)}}}}):j = 1,2,...,n\}  $$

In the above formula, *r*_*pea*_(·,·) is defined in formula 3, the definition of *u*^(*j*)^ and *v*^(*j*)^ :

$$\begin{aligned} {u^{(j)}} &= {({u_{1}},...,{u_{j - 1}},{u_{j + 1}},...,{u_{n}})^{T}},\\ {v^{(j)}} &= {({v_{1}},...,{v_{j - 1}},{v_{j + 1}},...,{v_{n}})^{T}} \end{aligned} $$

In above formula, *j*=1,2,…,*n*.

### Network reconstruction

Wang X [19] proposed the small world and scale-free network characteristics of complex networks such as PPI networks. Goldberg D S [20] et al. proposed the concept of edge-based mutual clustering coefficient based on the small world network characteristics of the PPI network to quantify the network structure. After calculating the MCC values of all edges in the network, setting a threshold and selecting a reliable structure which above the set threshold. Samanta MP [21] et al. found through experiments that if the number of adjacent junctions where two proteins act together is large, they have a close functional relationship. Segura J et al. [22] proposed a new method of using neighborhood cohesion to infer the interaction between protein interaction networks. Experimental results show that this method has good performance and can effectively predict PPI network interaction pairs. Based on those, we use topology coefficient *PTC* as a quantitative representation of PPI network topological structure feature *PTC* is obtained by parameter *α* adjustment with topological coefficient *T*(*u*,*v*) which representing the number of neighboring nodes of a node and a clustering factor *C*_*n*_ which representing the sharing of interaction nodes with other nodes. The calculation formula of *PTC* is shown in formula 5.

Combining the similarity of the *PTC* representing the network topology with gene expression patterns, the weight *w*(*u*,*v*) of the protein interaction pair in the PPI network is re-assigned and defined as the product of *T*(*u*,*v*) and *GEC*(*u*,*v*), as shown in formula 6:

5 $$ PTC(u,v) = \alpha {C_{n}} + (1 - \alpha)T(u,v)  $$

6 $$ \omega (u,v) = PTC(u,v)*GEC(u,v)  $$

The weight *w*(*u*) of node *u* is presented by the sum of node *u* and its edge in the PPI network, the formula is as follow:

7 $$ \omega (u) = \sum\limits_{(u,v) \in E} {\omega (u,v)}  $$

In the networks, the clustering factor indicates the strength of the connecting edges between the neighboring nodes of a node, and the topology factor indicates the strength of the neighboring nodes of the node. The clustering factor and the topological factor are assigned weights through parameters and combined, then the topological structure of the network can be fully expressed. *PTC* measures the density of adjacent nodes between a node and its neighboring nodes, and the value of the coefficient ranges from 0 to 1.The larger the *PTC* value, the more likely the neighboring nodes of the node will appear in the same cluster. *GEC* represents the corresponding gene expression similarity of protein interaction pair, that is, gene expression correlation measures the correlation between two proteins, and its value is between -1 and 1,the higher the *GEC* value, the higher the degree of protein co-expression, the greater the probability of appearing in the same functional module. Therefore, we weight the protein interaction by combining the topological structure of the PPI network and the correlation of gene expression, and the network distance between two nodes is a re-weighting of the topological distance in the network. Comprehensively consider *PTC* and *GEC* to calculate the probability that a node and its neighbor nodes appear in a cluster.

After integrating the topological coefficient *PTC* of the PPI network and the gene expression correlation *GEC* to calculate the *w* of all nodes in the graph, sorting *w* value of all nodes, and then choosing the highest weight as starting point.

### Algorithm description

**Fig. 1:**
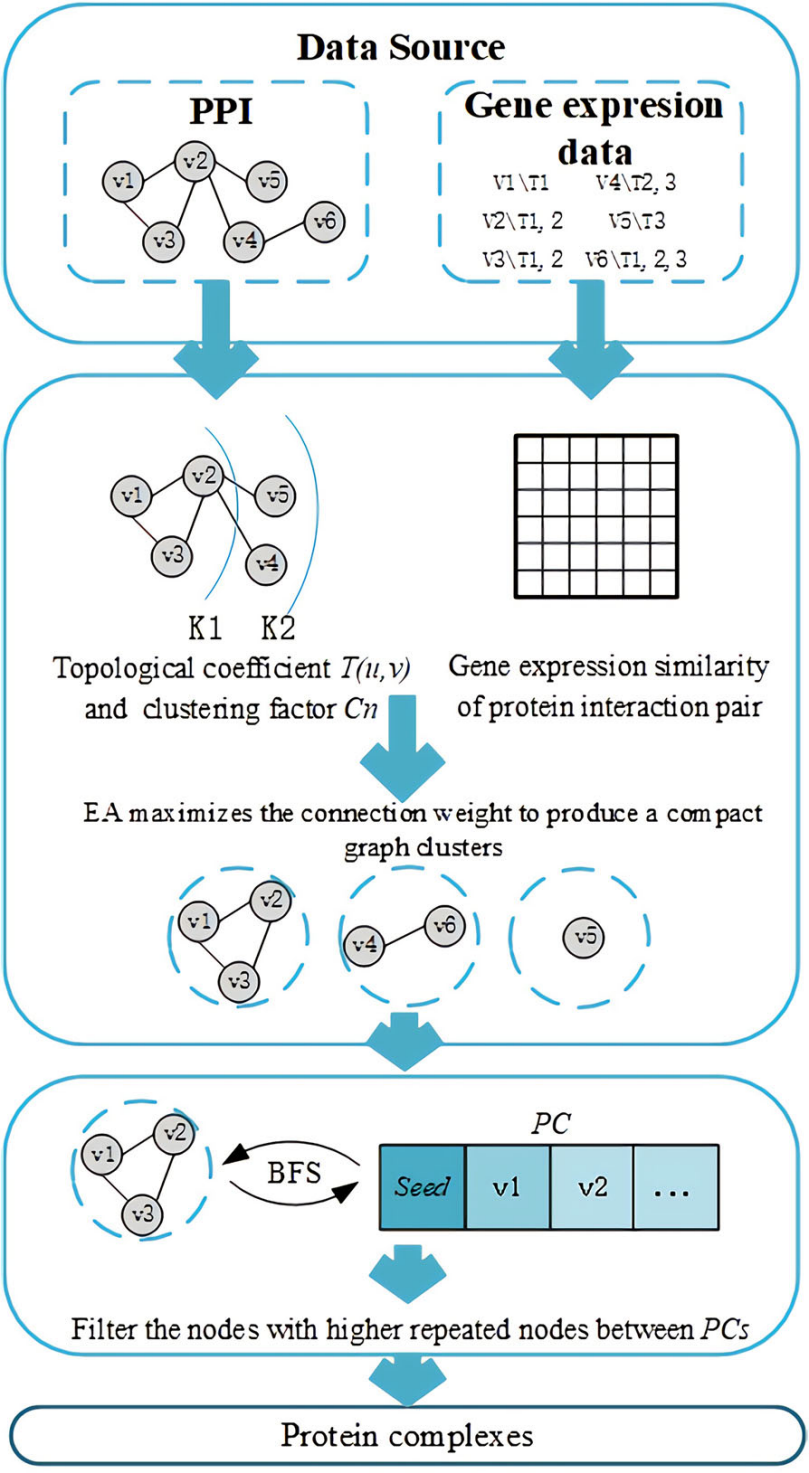
Schematic overview of our proposed ECTG model

Figure 1 shows the ECTG process, ECTG decomposes the PPI network into closely connected subgraphs to detect functional modules. The process is mainly divided into four steps. The first step is to construct a PPI network diagram with attributes based on the PPI network and gene expression data. The second step is to construct a weighted attribute PPI graph using *PTC* and *GEC*, given the attributed PPI network graph obtained in the first step, ECTG determines the weight of each edge in the graph according to the topological coefficient and the similarity of gene expression. In the third step, given a weighted graph, EA maximizes the connection weight to produce a compact graph clusters. In the fourth step, given graph clusters, a breadth-first search strategy is adopted, and searching subgraphs in each graph cluster according to the homogeneity of the attribute values of the connected nodes. The vertices of these subgraphs have similar attribute values and are relatively dense, and have a good correspondence with protein complexes in real life.

ECTG searches PPI pairs with higher values in each subgraph, and then continuously absorbs seed nodes to form modules. After ECTG has calculated all the values of *w* in the PPI network, the breadth-first search method BFS (breadth-first search) is used to extend the seeds, and form a protein complex finally. BFS can be divided into two stages, the first step: select an edge with the maximum *w* value *w*_*max*_ first, and then incorporate the two end points *v*_*i*_ and *v*_*j*_ connecting the edge into the seed node set of a protein complex; the second step: on the basis of *w*_*max*_, search for all adjacent nodes of *v*_*i*_ and *v*_*j*_ and extend all the nodes whose *w* value is greater than the threshold *λ* into the protein complex. The extended node definition is shown in formula 8:

8 $$ e(seed:{v_{k}}) = \left\{ \begin{array}{l} e \cup {v_{m}}{\mathrm{ }}\qquad if\ {\mathrm{ }}{w_{km}} \ge \lambda \\ e \cup \Phi {\mathrm{ }}\qquad otherwise \end{array} \right.  $$

In the above formula, *v*_*k*_ represents the node in the seed set, and *v*_*m*_ represents the node adjacent to the node *v*_*k*_. Only points whose *w* value is greater than the threshold can be merged into the set. The second stage of the search process will continue until no new nodes are added to the seed set. When a cluster completes the above search, ECTG will use the protein in the seed set to form a protein complex. Until all nodes are traversed, ECTG stops absorbing nodes. Due to the high probability of appearing small-scale modules using the above search strategy, ECTG will delete those modules that have been identified as containing less than 3 nodes. In order to reduce the redundancy of proteins in the recognition module, ECTG calculates the overlap score between any module and all others. The definition of overlap score is shown in formula 9:

9 $$ O{v_{r}} = \max \frac{{\left| {e \cap P{C_{I}}} \right|}}{{\left| {e \cup P{C_{I}}} \right|}}  $$

where *e* and *PC*_*I*_ respectively refer to the module obtained after a search and any other modules in the result set. ECTG then uses a threshold *OvMax* to exclude those modules whose overlap score is higher than the threshold. In order to explain the ECTG method in more detail, we give its pseudo code, as shown in Algorithm 1.

The input information of ECTG includes: PPI network, gene expression data, parameter *α* used to control the weight of topological coefficients, used to filter out threshold *λ* that do not meet similarity, and used to filter the nodes with higher repeated nodes between the obtained modules.


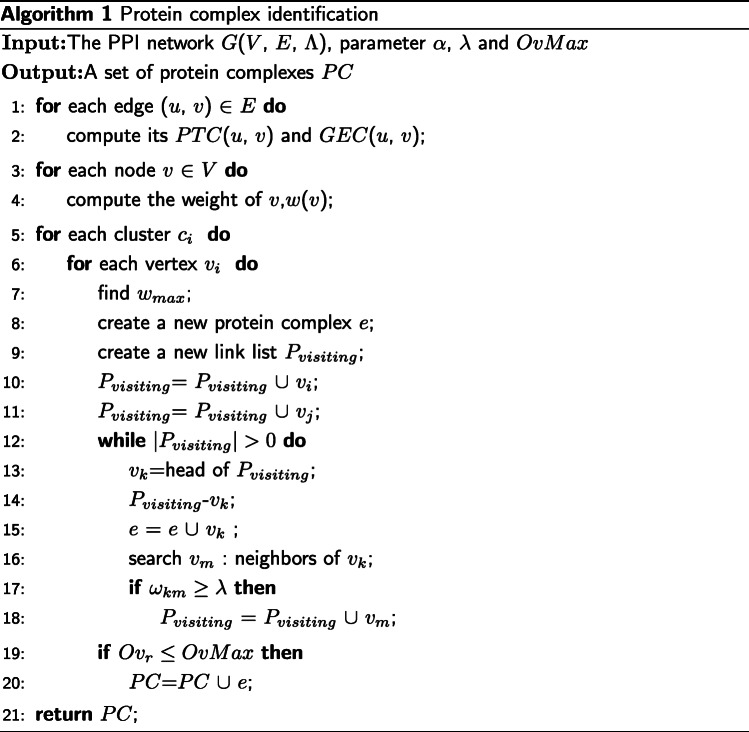


## Results and analysis

### Experimental data set

The experimental process is to link the PPI network and gene expression, and apply the ECTG algorithm to the Saccharomyces cerevisiae data set, which is downloaded from the 2013 version of the DIP database. The network contains 4579 points and 20845 edges after process. And the Krogan and Gavin data sets, the specific information is shown in Table 1. Obviously, there are great differences of the datasets in the number of proteins and protein-protein interactions. This can increase the credibility of the results obtained by ECTG algorithm and prove to have better generalization ability of propose algorithm. The gene expression data is selected from the publications of Rintala et al. [23], this gene expression data is the data sequence of yeast response to sudden hypoxia [17], that is, the glucose-limited cultivation analysis after the transition from fully aerobic (20.9% *O*_2_ or restricted oxygen (1.0% *O*_2_) to anaerobic state. 79 hours (20.9% *O*_2_) or 72 hours (1.0% *O*_2_) after shifting. These data provide insights into the adaptive mechanism of the transition from respiration to fermentation growth. After processing, the gene expression data has 5664 unique non-empty genes, and each gene expression includes 28 time courses. Comparing the two information, there are 4936 proteins in PPI network and 4616 proteins have gene expression.

**Table 1:** Datasets

Datasets	Number of protein	Number of interactions
DIP	4930	17201
Krogan	3581	14076
Gavin	1430	6531

### Experimental design

When testing method performance, ECTG is compared with different algorithms, including ClusterONE [24], DPClus [25], COACH [26] and CFinder [27]. We use these five methods to detect functional modules in the above three data sets. ClusterONE, DPClus, COACH and CFinder detecting functional modules only based on the topological structure of the PPI network, not make full use of node attribute information. Such as MCL, ClusterONE can be used for weighted PPI network data, which can be compared with the method ECTG using a weighted network. For the above methods, their respective parameter settings are shown in Table 2.

**Table 2:** Parameter settings of different algorithms

Algorithm	Parameter
ClusterONE	*s*=3, *density*=auto(default setting)
DPClus	*CPin*=0.5, *din*=0.6(default setting)
COACH	*W*=0.225(default setting)
CFinder	*k*=3
MCL	*inflation*=1.8(default setting)
ECTG	*α*=0.8,*λ*=0.7/0.8,*OvMax*=0.7/0.8/0.9

### Method performance analysis

Table 3 summarizes the indicators obtained by executing different algorithms. On the DIP data set, the accuracy of ECTG is 0.49, which is slightly lower than that of the MCL algorithm, but its recall rate is 0.65, which is much higher than that of MCL, and its F-measure is also about 15% higher than other methods. The situation is similar on the Gavin and Krogan data sets. ECTG obtained the best F-measure values on the 3 data sets. Although ECTG has not always obtained the best Precision and Recall values, has always obtained better F-measure values than other methods, indicating that the performance of this method for detecting functional modules is better than other methods. At the same time, the algorithm results will be affected by the difference of datasets. ECTG can always maintain advanced performance on one or more indexes on three data sets. From experimental results we can conclude that the functional modules obtained by the ECTG method may more accurately represent the real modules in the standard set and have better generalization ability. Regarding the size and coverage of the detected modules, the number of modules identified by ECTG in each set of data is relatively small compared to MCL, the false positives are low, and the coverage is relatively large, so its coverage is relatively high. In order to check whether other algorithms obtain the same or better performance when using the same weighted PPI network data, we compare the results of those algorithms that can process weighted network data, including ClusterONE and MCL. The results are shown in Table 4. As shown in the table, ECTG’s accuracy rate is 0.68 on the Gavin data set, which is slightly lower than the MCL algorithm, but the Recall has increased by nearly 20%, so its F-measure value has increased by about 15% compared with the other two algorithms. When dealing with weighted networks, ClusterONE and MCL use weighted network data generated by combining topology and gene expression data, the performance has varying degrees of improvement. But ECTG is still superior to these two algorithms, and the results show that considering the topological and attribute factors, ECTG’s performance is better than the algorithm that only considers the network topology. In short, ECTG performs better in detecting functional modules. It obtains better F-measure results in most data sets. The result is affected by the difference of data sets, but ECTG can always maintain advanced performance on one or more indicators.Therefore, ECTG can achieve better results when regard the task of functional module detection as the problem of considered gene expression data and topology optimization.

**Table 3:** Results of CR, precision, Recall and F-measure

Data Set	Algorithms	Number of PC	CR	Precision	Recall	F-measure
Gavin	ECTG	297	0.38	0.68	0.57	0.62
	ClusterONE	245	0.44	0.39	0.37	0.38
	DPClus	219	0.37	0.40	0.36	0.38
	COACH	326	0.32	0.42	0.45	0.43
	CFinder	99	0.24	0.53	0.19	0.28
	MCL	121	0.31	0.72	0.33	0.45
Kroga	ECTG	518	0.54	0.55	0.66	0.6
	ClusterONE	241	0.59	0.49	0.41	0.45
	DPClus	495	0.3	0.26	0.49	0.34
	COACH	349	0.48	0.48	0.54	0.51
	CFinder	113	0.46	0.48	0.22	0.3
	MCL	371	0.47	0.63	0.09	0.16
DIP	ECTG	436	0.68	0.49	0.65	0.56
	ClusterONE	337	0.38	0.42	0.36	0.39
	DPClus	843	0.44	0.21	0.63	0.31
	COACH	849	0.56	0.35	0.63	0.45
	CFinder	189	0.65	0.38	0.19	0.25
	MCL	396	0.52	0.59	0.20	0.29

**Table 4:** Experimental results using weighted network data

Data Set	Algorithms	Number of PC	CR	Precision	Recall	F-measure
Gavin	ECTG	297	0.38	0.68	0.57	0.62
	ClusterONE	155	0.32	0.59	0.36	0.44
	MCL	146	0.34	0.73	0.35	0.47
Krogan	ECTG	518	0.54	0.55	0.66	0.6
	ClusterONE	221	0.55	0.50	0.43	0.46
	MCL	412	0.53	0.64	0.18	0.27
DIP	ECTG	436	0.68	0.49	0.65	0.56
	ClusterONE	239	0.38	0.42	0.36	0.39
	MCL	382	0.56	0.61	0.23	0.33

### Parameter settings

**Fig. 2:**
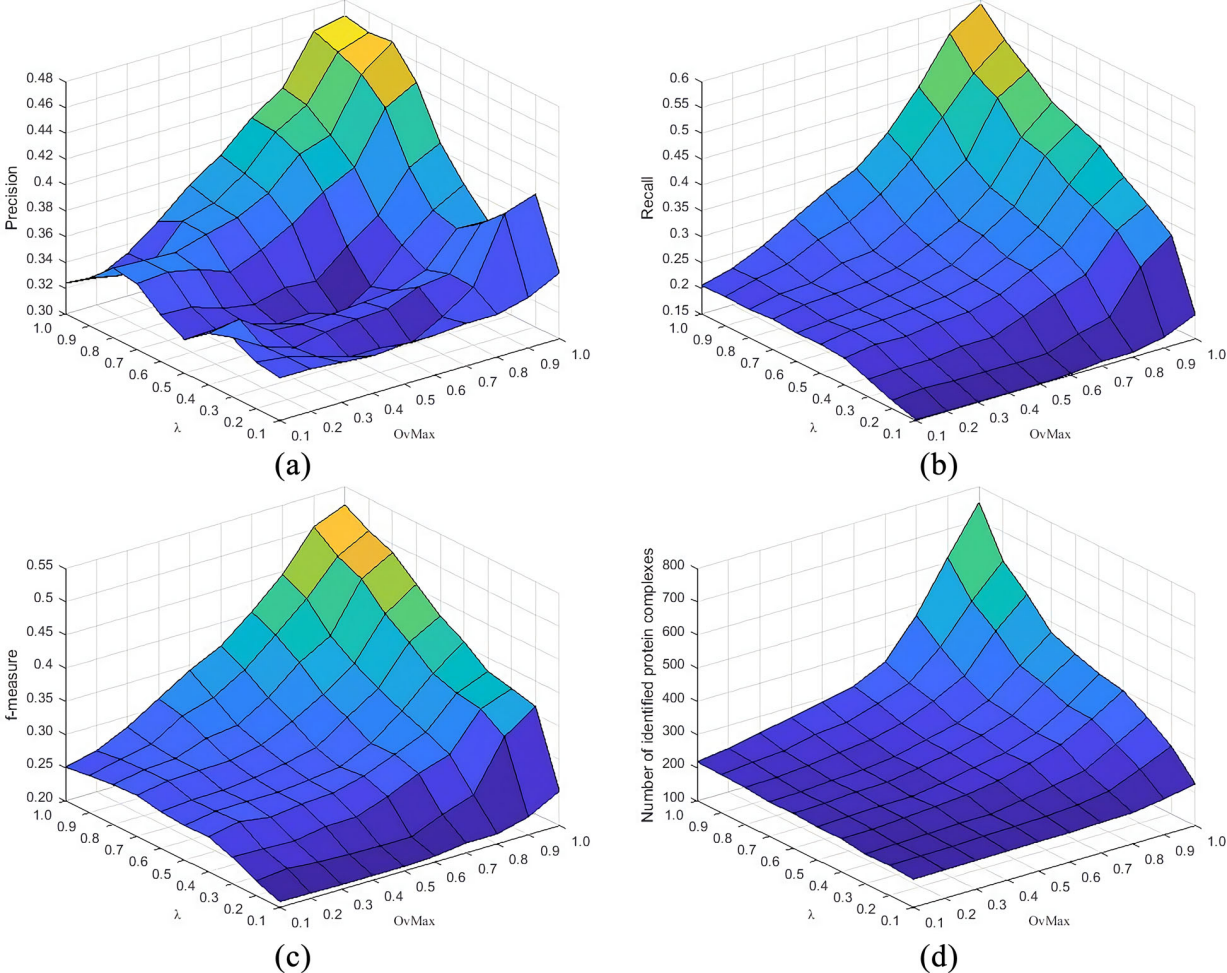
Results of precision, Recall, F-measure and the number of protein complexes identified by ECTG using *α*=0.2 and different settings of *λ* and *OvMax*

**Fig. 3:**
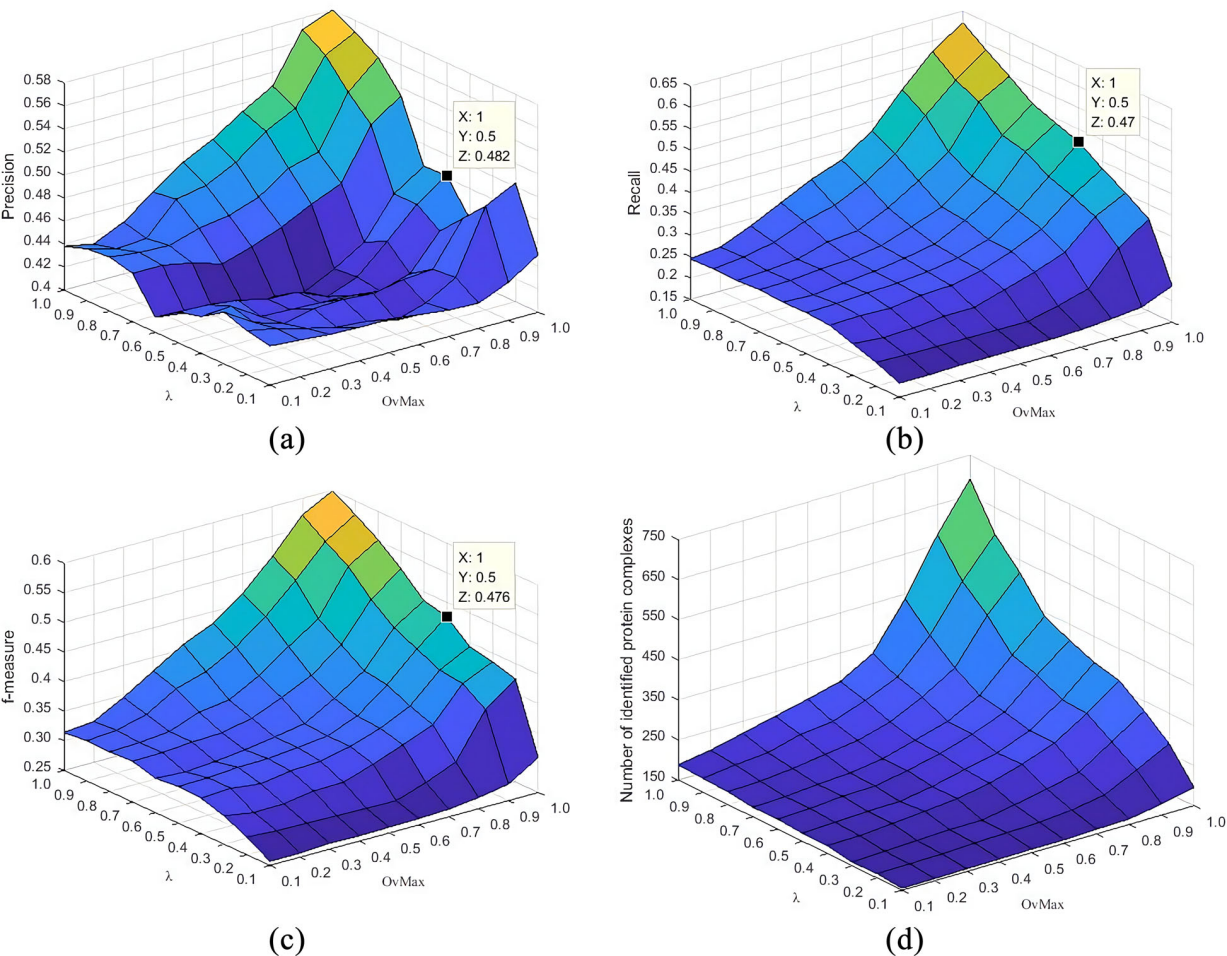
Results of precision, Recall, F-measure and the number of protein complexes identified by ECTG using *α*=0.5 and different settings of *λ* and *OvMax*

**Fig. 4:**
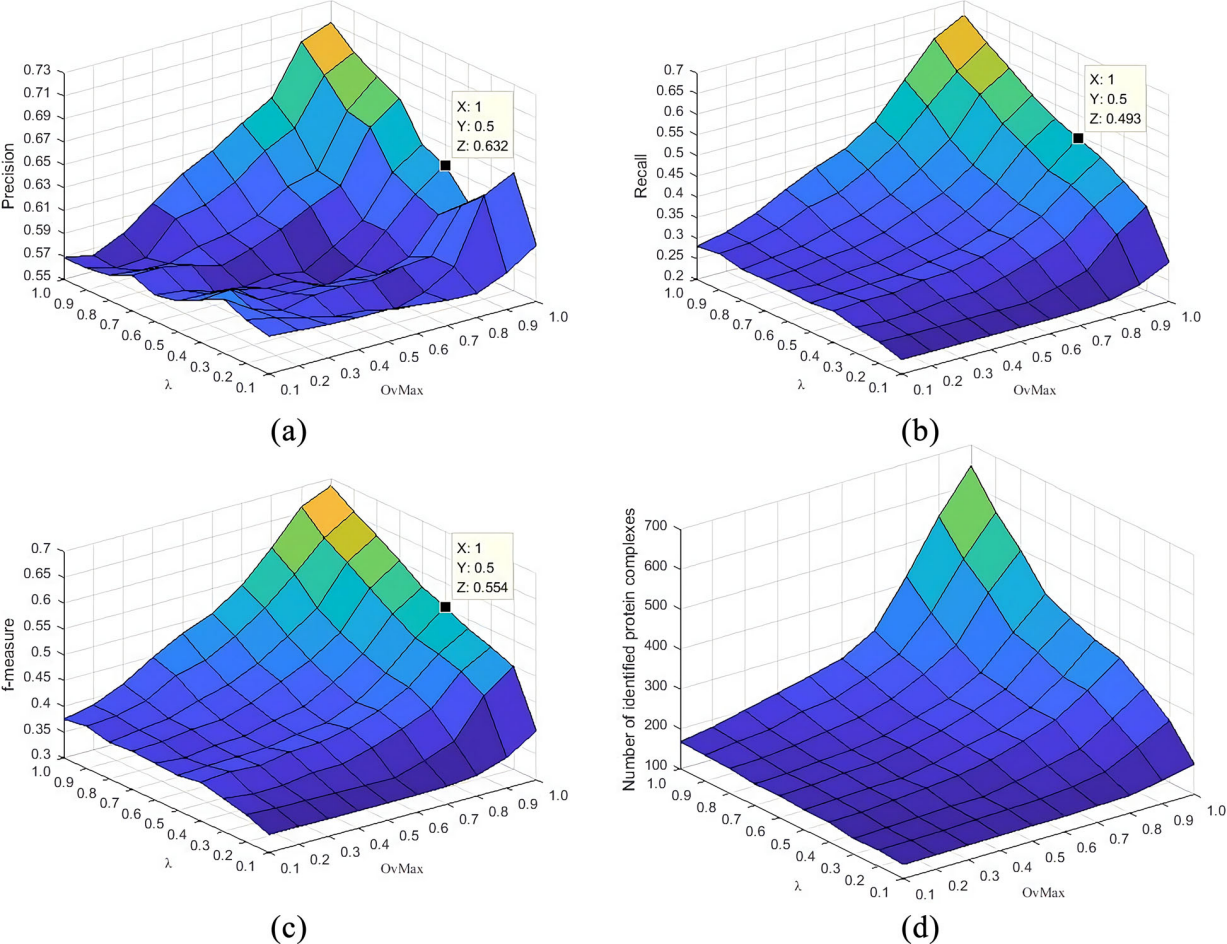
Results of precision, Recall, F-measure and the number of protein complexes identified by ECTG using *α*=0.8 and different settings of *λ* and *OvMax*

As mentioned earlier, there are three parameters in the ECTG execution process that determine the result of the detection module: *α*,*λ* and *OvMax*. In order to understand how these parameters affect the experimental results, we change *α*,*λ* and *OvMax* from 0.1 to 1 in steps of 0.1 to detect modules using above three PPI network data. After collecting the experimental results under different parameter combinations, we evaluated the evaluation indexes of Precision, Recall and F-measure. The Figs. 2, 3 and 4 show the changes of different parameters of the Gavin data set, listing the impact of changes in *λ* and *OvMax* when *α* respectively equal 0.2, 0.5 and 0.8 on the evaluation index. After analyzing the results of multiple experiments, obtain the changes in evaluation index when *α* equal 0.2, 0.5 and 0.8 respectively. It can be seen from figures that overall precision value, recall value and F-measure increased by about 12%, 8% and 7% respectively when *α* equal 0.5 than *α* equal 0.2. But the number of protein complexes decreased by nearly 50. Comparing with *α* equal 0.5 When *α* equal 0.8, the precision value increased by about 14%, the recall value increased by nearly 4%, the F-measure value increased by about 9%, and the number of protein complexes decreased by nearly 20. As *α* increases, the value of the index is also increasing, and the increment in the range of 0.1-0.5 is lower than the increment in the range of 0.5-1.0. Although the value obtained near *α* equal 1.0 is relatively high, many complexes that actually exist but do not meet the filter conditions are filtered out, so that the number of modules is relatively small, the Recall value is relatively increased, and the F-measure value is relatively increased. This will omit part of the real modules, which is not the best experimental result. Therefore, the best value of *α* in this experiment is 0.8.

Shown in Fig. 4a-c, when *α* equal 0.8, the changing trends of precision and F-measure are similar when *λ* and *OvMax* change, simply setting *λ* and *OvMax* near 0 or 1, the obtained results are not optimal. For example, when *λ* is set to 0.2, no matter how you adjust the value of *OvMax*, the precision obtained by ECTG is a relatively low value. When a smaller value is used, ECTG includes more nodes with lower similarity, resulting in a larger gap between the clustered modules and the real modules. Although when *λ* and *OvMax* are set near 1, ECTG cannot identify those modules that contain more nodes so that some real modules are lost. Considering these conditions, it is necessary to set appropriate values of *λ* and *OvMax* for the experimental performance of the ECTG method. As shown in Fig. 4d, ECTG can identify more modules in the PPI network with higher *λ* and *OvMax* values, so this method can obtain more protein complexes in the standard set and achieve a higher recall value.

Therefore, we expect a method to accurately detect relatively more nodes. In general, we recommend that the values of *λ* and *OvMax* are between 0.6 and 0.9 when the ECTG detects the module. When *λ* and *OvMax* is properly set in this range, ECTG may perform better. This is why we used the parameter settings shown in Table 2 in the ECTG experiment.

### Functional enrichment analysis

**Fig. 5:**
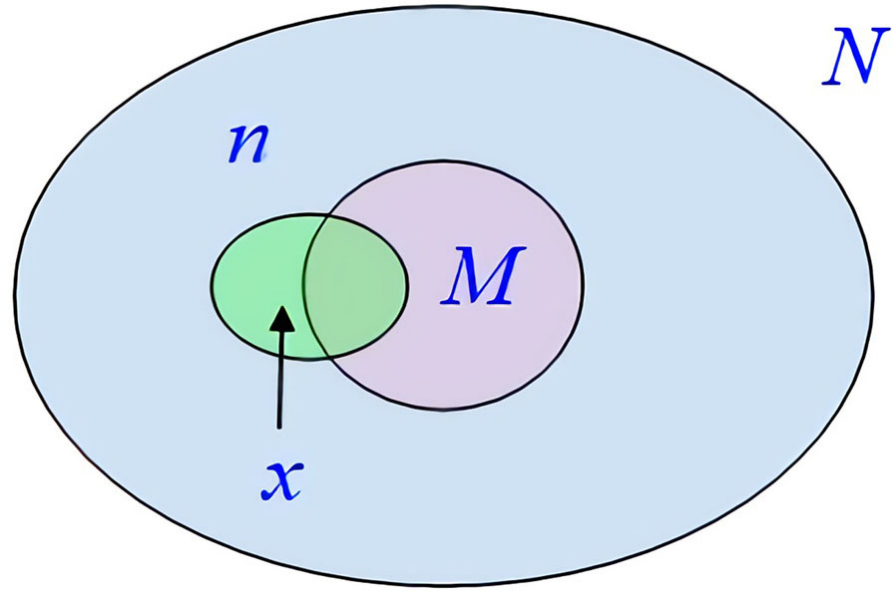
The definition of *P*-value

The probability of functional homology of actual protein functional modules is very high. This part uses the three kinds of annotation information contained in the GO database [28] and GO: TermFinder to calculate the *P*-value of the module obtained by the algorithm to determine its biological function significance [29], and mark it’s functional annotations, so the *P*-value [30] of inside modules protein co-occurrence probability need be calculated. The concept of *P*-value is described as follows: given ontology *d*, we use *N* to represent the protein quality annotated in ontology *d*. Given *a* notes, we will denote the total number of proteins covering *a* by *M*. Given a cluster *b*, *n* represents the number of protein contained therein, and *x* represents the number of proteins with *a* annotations in it. When the ontology *d* and the term *a* are randomly given, the probability that the number of proteins is greater than or equal to *x* in *b* is represented by *P*-value. The definition is shown in Fig. 5, and the calculation method is shown in formula 10:

10 $$ p - value = {\sum\nolimits}_{i = x}^{n} {\frac{{\left(\begin{array}{l} M\\ i \end{array} \right)\left(\begin{array}{l} N - M\\ n - i \end{array} \right)}}{{\left(\begin{array}{l} N\\ n \end{array} \right)}}}  $$

In order to verify the effectiveness of ECTG, we calculated the *P*-values of all modules on the DIP network by comparing the probability of actual exist modules obtained by various algorithms. Using Go: TermFinder, a web-based service that can search for important shared GO items in the obtained module proteins. In our experiment, the threshold range of *P*-value is set from 1E-15 to 1E-2. That means those GO items whose *P*-value is less than or equal to the threshold are considered to be meaningful. Not all modules with significant GO items have been discovered, that is, they can be found in such as MIPS/CYGD and CYC2008, but they can be considered as true module candidates because of their functional enrichment analysis. After obtaining the *P*-value of each module, we count protein complexes in the detected modules that contain at least one GO item with a *P*-value lower than different thresholds.

**Fig. 6:**
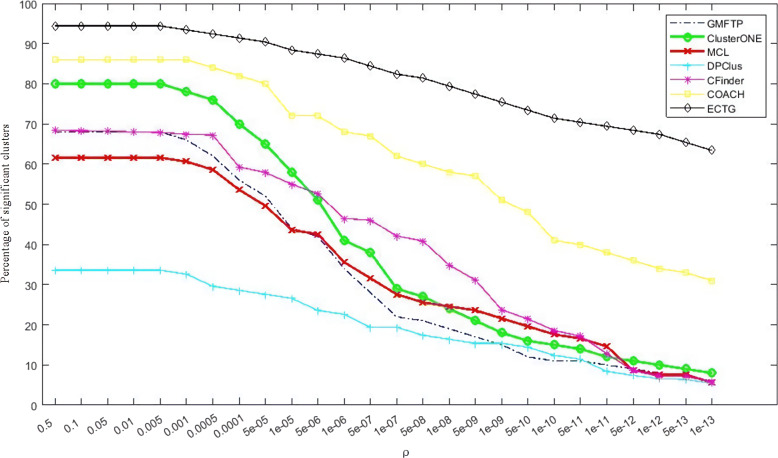
Percentage of significant clusters for various values of *P*-value cut-off calculated based on the BP domain

**Fig. 7:**
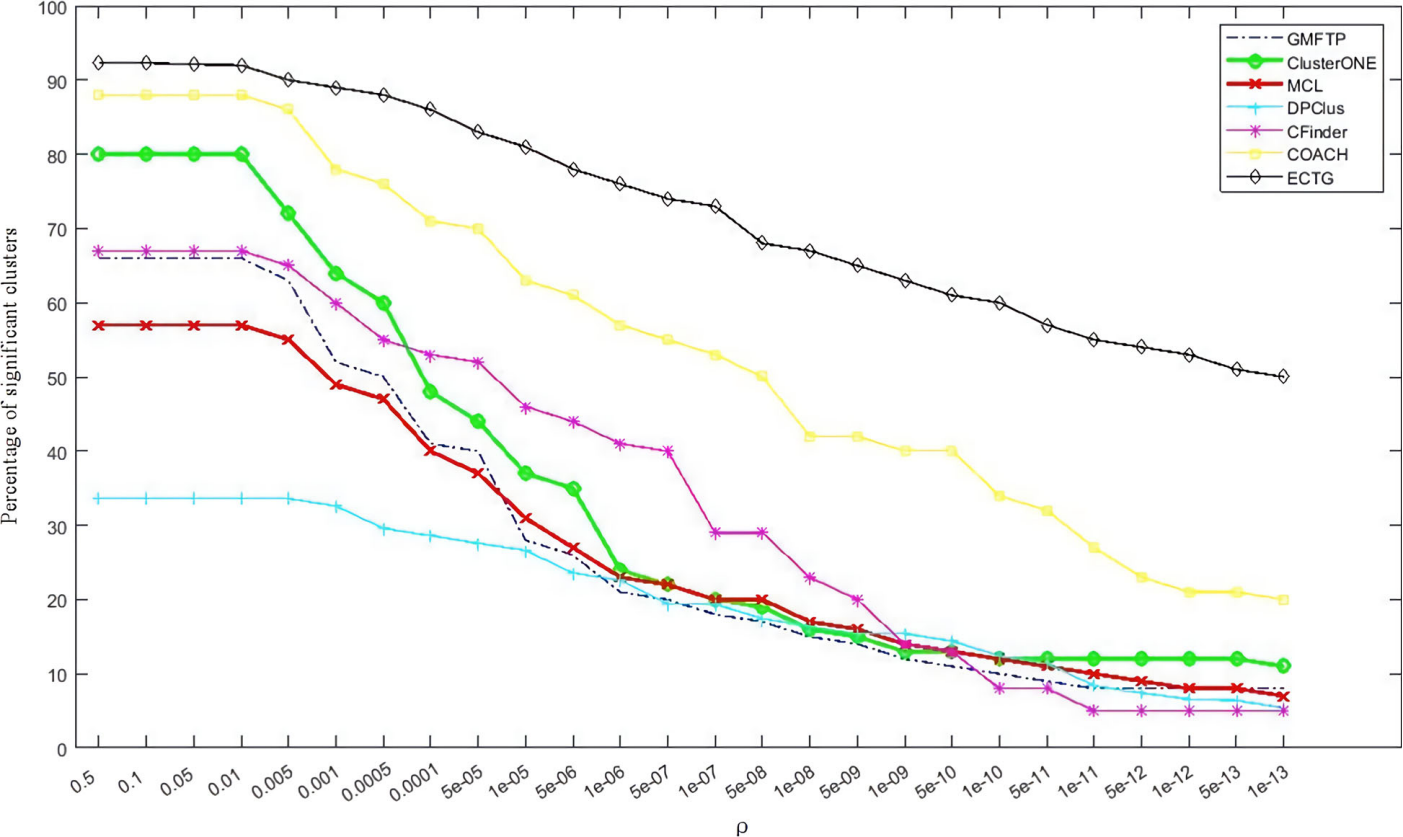
Percentage of significant clusters for various values of *P*-value cut-off calculated based on the MF domain

In addition to analyzing the modules obtained through ECTG, we also calculated GMFTP, MCL, ClusterONE, CFinder, DPClus and COACH. GMFTP has proven to be very effective when considering network topology and functionality. And MCL, IPCA, ClusterONE proved to be more effective for the recognition module after considering the topology. The above-mentioned methods are chosen as the comparison method of ECTG because they all show better robustness on the three data sets mentioned above. The experimental results of ECTG, GMFTP, MCL, ClusterONE, CFinder, DPClus and COACH are shown in Table 5. Obtained from the table: ECTG detects proteins with more significant GO items than other methods, especially when the *P*-value threshold is low, such as *P*-value < 1E-15. At the same time, perform specific GO biological process(BP) annotation and GO molecular function(MF) annotation analysis on the identified functional modules on the DIP data set. BP stands for a collection of molecular events that begin and end. These events are closely related to the functions of integrated life units (cells, tissues, organs, and organisms). Calculate the *P*-value based on the BP and MF domains of GO. In this experiment, if P < *ρ*, and *ρ* is the threshold of *P*-value, the predicted cluster P is significant. The figure shows the percentage of important clusters for several *ρ* values. Figures 6 and 7 show the *P*-values calculated by BP and MF respectively. It can be seen from the figure that the ECTG method obtains more clusters with lower *P*-values when detecting modules. This data shows that ECTG can detect more modules rich in biological significance than other methods. No matter how many protein complexes are currently known, they have a higher probability of becoming a real complex that is identified through biological experiments in the future. Based on the results of the *P*-value experiment, it can be seen that ECTG performs better when detecting functional modules, and is a better method for detecting and predicting protein functional modules.

**Table 5:** *P*-value test on protein complexes identified by different algorithms

Data Set	Algorithms	Average-log(*P*-value)	<1E-15	<1E-10	<1E-5	<1E-2
DIP	ECTG	12.3	34.88%	53.50%	83.31%	94.66%
	GMFTP	9.6	14.63%	27.9%	55.92%	76.79%
	MCL	6.67	7.36%	10.66%	31.07%	61.83%
	ClusterONE	7.99	11.12%	25.83%	58.76%	80.96%
	CFinder	6.85	5.16%	13.57%	35.24%	67.54%
	DPClus	4.45	0.78%	4.01%	12.35%	33.56%
	COACH	7.85	11.65%	24.06%	47.27%	82.36%
Krogan	ECTG	13.32	32.87%	49.78%	76.55%	89.64%
	GMFTP	8.70	17.52%	36.24%	64.25%	70.48%
	MCL	5.09	7.26%	13.79%	44.25%	69.21%
	ClusterONE	8.13	19.9%	36.7%	69.8%	87.4%
	CFinder	6.3	4.23%	11.04%	31.56%	58.90%
	DPClus	5.58	2.67%	5.98%	15.86%	36.82%
	COACH	9.25	15.86%	26.12%	49.25%	79.35%
Gavin	ECTG	13.67	43.52%	64.13%	85.86%	94.59%
	GMFTP	9.2	31.8%	52.86%	83.17%	92.13%
	MCL	6.23	23.56%	36.27%	65.08%	79.84%
	ClusterONE	8.26	21.6%	32.3%	61.7%	90.6%
	CFinder	5.96	3.89%	10.56%	29.56%	52.13%
	DPClus	6.03	5.17%	10.23%	20.59%	42.26%
	COACH	9.96	16.23%	27.26%	48.56%	78.64%

One of the protein functional modules obtained by the ECTG method, and the module size is more than 5 and the matching degree is more than 0.4, the topological structure and biological significance are analyzed as well, and 10 functional modules are selected for listing, as shown in Table 6. They not only have a lower *P*-value, but also have a higher consistency with known protein functional modules, and their *P*-value is smaller than 0.001.

**Table 6:** Complexes and its *P*-value detected on DIP dataset by ECTG

*P*-value	predicted complex	known complex	Biological
1.58e-35	YBR217W, YBR272C, YDL007W, YDL097C,	proteasome complex	ubiquitin-dependent
	YDL147W, YDR394W, YDR427W, YEL037C,		protein catabolic
	YER012W, YER021W, YFR004W, YFR010W,		process
	YFR052W, YGL004C, YGL048C, YHL030W,		
	YHR027C, YHR200W, YKL145W,YLR421C,		
	YMR314W, YOR117W, YOR259C, YOR261C,		
	YPR108W		
6.86e-17	YBL026W, YCR077C, YDR378C, YER112W,	spliceosomal	mRNA
	YER146W, YGL173C, YJL124C, YJR022W,	tri-snRNP	processing
	YKL173W, YLR147C,YLR275W, YMR268C,	complex	
	YNL118C, YPR178W, YOL149W, YNL147W,		
	YLR438C-A		
2.77e-27	YAL043C, YDR195W, YDR228C, YDR301W,	mRNA cleavage	mRNA
	YER133W, YGR156W, YJL033W, YJR093C,	factor complex	polyadenylation
	YKL018W, YKL059C, YKR002W, YLR115W,		
	YLR277C, YMR061W, YNL317W, YPR107C		
5.92e-18	YBR081C, YBR198C, YDR145W, YDR167W,	transcription factor	RNA polymerase
	YDR176W, YDR216W, YDR448W, YEL009C,	TFIID complex	II transcriptional
	YER148W, YGL112C, YGR274C, YML015C,		preinitiation
	YML098W, YMR236W		complex assembly
1.86e-18	YCR035C, YDL111C, YDR280W, YGR095C,	exosome	polyadenylation
	YGR158C, YGR195W, YHR069C, YNL189W,	(RNase complex)	-dependent
	YNL232W, YOL021C, YOR001W, YOR076C		snoRNA
			3’-end processing
4.46e-25	YBL093C, YBR193C, YBR253W, YDL005C,	core mediator	positive regulation
	YER022W, YGR104C, YHR041C, YHR058C,	complex	of transcription from RNA
	YOL051W, YOL135C, YPL248C		polymerase II promoter
4.03e-17	YBR055C, YDR473C, YPR178W, YGR091W,	U4/U6 x U5	mRNA splicing,
	YOR308C, YHR165C, YJR022W, YKL173W,	tri-snRNP complex	via spliceosome
	YLR147C, YLR438C-A, YFL017W-A		
1.58e-13	YAL021C, YCR093W, YDL165W, YER068W,	CCR NOT core	positive regulation of
	YGR134W, YIL038C, YNL288W, YNR052C,	complex	transcription elongation
	YPR072W		from RNA polymerase
			II promoter
3.14e-16	YDL232W, YEL002C, YGL022W, YJL002C,	oligosacchar	protein N-linked
	YMR149W,YGL226C-A, YOR085W, YOR103C	yltransferase complex	glycosylation
1.12e-09	YBR079C, YDR429C, YLR192C, YMR146C,	translation	formation of translation
	YNL244C, YOR361C, YPR041W, YPR086W	preinitiation complex	preinitiation complex

## Algorithm prediction example analysis

**Fig. 8:**
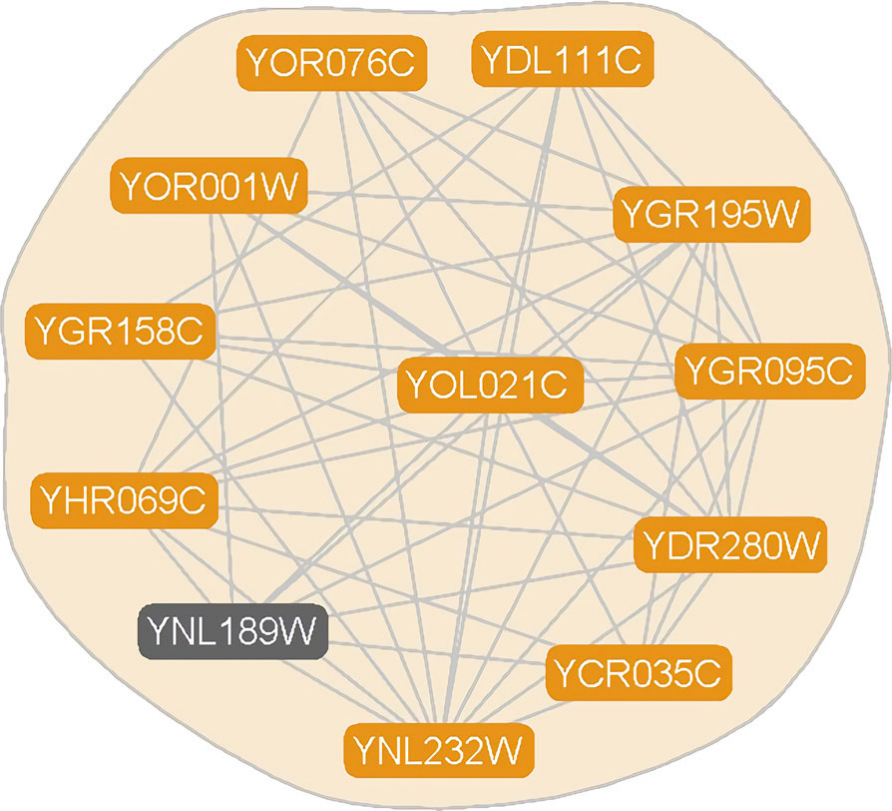
Example of predicted clusters

The experimental results show that the functional modules predicted by combining the topological structure of the PPI network and gene expression data can match more modules rich in biological functional significance, and provide beneficial help for predicting protein functional modules and the proteins whose functions have not yet been revealed in the modules. As shown in Fig. 8, the ECTG detection scale is 12 modules, of which 11 belong to the molecular functional group heterocyclic compound binding, so the protein YNL189W may also have this function.

## Conclusion

This paper proposes a functional module detection method ECTG that combines topological structure and gene expression data. The idea is expressing the topological structure of the PPI network with quantified topological features, and then using the gene expression data to calculate the similarity of gene expression patterns. Combining the two to re-weight the PPI pairs in the network, reconstructing the PPI network, and finally performing clustering on the PPI network through the EA algorithm. First, the reason and goal of introducing gene expression data and topology structure are explained. Second, the ECTG clustering algorithm is described in detail. Finally, perform clustering experiments on three different yeast mutual data sets to detect functional modules. The analysis of experimental results shows that compared with other methods, the clustering algorithm proposed in this paper has a greater improvement in recall rate and Fmeasure value, which shows that the combination of topological structure and gene expression data is effective. The advantages of ECTG are reflected in two aspects: (1) It can effectively remove the noise data reflected by calculating the topological structure feature value in the PPI network through the similarity of gene expression patterns; (2) using the information hidden in the gene expression data appropriately.

## Data Availability

The GO is freely available from http://geneontology.org/docs/ontology-documentation/.

## Abbreviations

PPI: Protein-protein interaction
EA: Evolutionary algorithm
BFS: Breadth-first search
PC: Protein complexes

## References

[1] Shen H, Cheng X, Cai K, Hu MB (2008). Detect overlapping and hierarchical community structure in networks. Physica A Stat Mech Appl.

[2] Li M, Wang J, Chen J. A fast agglomerate algorithm for mining functional modules in protein interaction networks. In: International Conference on Biomedical Engineering and Informatics: 2008. p. 3–7.

[3] Qing-sheng HU, Xiu-juan LEI (2015). Improved MCL clustering algorithm in PPI networks. Comput Sci.

[4] Gu L, Han Y, Wang C, Chen W, Jiao J, Yuan X (2019). Module overlapping structure detection in PPI using an improved link similarity-based Markov clustering algorithm. Neural Comput & Applic.

[5] Yuan X, Xie L, Abouelenien M (2018). A regularized ensemble framework of deep learning for cancer detection from multi-class, imbalanced training data. Pattern Recogn.

[6] Xia Y, Wang X, Gu L, Gao Q, Jiao J, Wang C (2020). A collective entity linking algorithm with parallel computing on large-scale knowledge base. J Supercomput.

[7] Chin CH, Chen SH, Ho CW, Ko MT, Lin CY (2010). A hub-attachment based method to detect functional modules from confidence-scored protein interactions and expression profiles. BMC Bioinformatics.

[8] Zhao B, Wang J, Li M, Li X, Li Y, Wu FX, Pan Y (2016). A new method for predicting protein functions from dynamic weighted interactome networks. IEEE Trans Nanobiosci.

[9] Rhrissorrakrai K, Gunsalus KC (2011). Mine: Module identification in networks. BMC Bioinformatics.

[10] Zuo Y-C, Su WX, Zhang SH, Wang SS, Wu CY, Yang L, Li GP (2015). Discrimination of membrane transporter protein types using K-nearest neighbor method derived from the similarity distance of total diversity measure. Mol BioSyst.

[11] Yuan X, Buckles BP, Yuan Z, Zhang J. Mining negative association rules. In: Proceedings ISCC 2002 Seventh International Symposium on Computers and Communications: 2002. p. 623–8.

[12] Zhao B, Wang J, Li M, Wu FX, Pan Y (2014). Detecting protein complexes based on uncertain graph model. IEEE/ACM Trans Comput Biol Bioinforma.

[13] Butz M, Steenbuck ID, Ooyen AV (2014). Homeostatic structural plasticity increases the efficiency of small-world networks. Front Synaptic Neurosci.

[14] Gu L, Wang C, Zhang Y, Zhong J, Ni Z (2014). Trust model in cloud computing environment based on fuzzy theory. Int J Comput Commun Control.

[15] Rao H, Shi X, Rodrigue AK, Feng J, Xia Y, Elhoseny M, Yuan X, Gu L (2019). Feature selection based on artificial bee colony and gradient boosting decision tree. Appl Soft Comput.

[16] Xenarios I, Salwinski L, Duan XJ, Higney P, Kim S-M, Eisenberg D (2002). DIP, the Database of Interacting Proteins: a research tool for studying cellular networks of protein interactions. Nucleic Acids Res.

[17] Krogan N, Cagney G, Yu H, Zhong G, Guo X, Ignatchenko A, Li J, Pu S, Datta N, Tikuisis A (2006). Global landscape of protein complexes in the yeast Saccharomyces cerevisiae. Nature.

[18] Gavin AC, BeSche M, Krause R, Grandi P, Marzioch M, Bauer A, Schultz J, Rick JM, Michon AM, Cruciat CM (2002). Functional organization of the yeast proteome by systematic analysis of protein complexes. Nature.

[19] Wang XF, Chen G (2003). Complex networks: Small-world, scale-free and beyond. IEEE Circ Syst Mag.

[20] Goldberg DS, Roth FP (2003). Assessing experimentally derived interactions in a small world. Proc Natl Acad Sci U S A.

[21] Samanta MP, Liang S. Proc Natl Acad Sci U S A. 2003; 100(22):12579–83. 10.1073/pnas.2132527100 PMC240660 14566057

[22] Joan S, Sorzano COS, Jesus CA, Patrick A, Carazo JM (2015). Using neighborhood cohesiveness to infer interactions between protein domains. Bioinformatics.

[23] Rintala E, Jouhten P, Toivari M, Wiebe MG, Maaheimo H, Penttil M, Ruohonen L (2011). Transcriptional responses of Saccharomyces cerevisiae to shift from respiratory and respirofermentative to fully fermentative metabolism. Omics J Integr Biol.

[24] Nepusz T, Yu H, Paccanaro A (2012). Detecting overlapping protein complexes in protein-protein interaction networks. Nat Methods.

[25] Altaf-Ul-Amin M, Shinbo Y, Mihara K, Kurokawa K, Kanaya S (2006). Development and implementation of an algorithm for detection of protein complexes in large interaction networks. Bmc Bioinformatics.

[26] Wu M, Li X, Kwoh CK, Ng SK (2009). A core-attachment based method to detect protein complexes in PPI networks. BMC Bioinformatics.

[27] Adamcsek B, Palla G, Farkas IJ, Derenyi I, Vicsek T (2006). CFinder: locating cliques and overlapping modules in biological networks. Bioinformatics.

[28] Ashburner M, Ball CA, Blake JA, Botstein D, Butler H, Cherry JM, Davis AP, Dolinski K, Dwight SS, Eppig JT (2000). Gene ontology: tool for the unification of biology. Nat Genet.

[29] Boyle EI, Weng S, Gollub J, Jin H (2004). GO:: TermFinder?open source software for accessing Gene Ontology information and finding significantly enriched Gene Ontology terms associated with a list of genes. Bioinformatics.

[30] Maraziotis IA, Dimitrakopoulou K, Bezerianos A (2007). Growing functional modules from a seed protein via integration of protein interaction and gene expression data. BMC Bioinformatics.

